# DRAMP: a comprehensive data repository of antimicrobial peptides

**DOI:** 10.1038/srep24482

**Published:** 2016-04-14

**Authors:** Linlin Fan, Jian Sun, Meifeng Zhou, Jie Zhou, Xingzhen Lao, Heng Zheng, Hanmei Xu

**Affiliations:** 1 School of Life Science and Technology, China Pharmaceutical University, Nanjing 210009, China; 2 MOE Key Lab of Bioinformatics, School of Life Sciences, Tsinghua University, Beijing 100084, China; 3 Center of Drug Discovery, State Key Laboratory of Natural Medicines, China Pharmaceutical University, Nanjing 210009, China

## Abstract

The growing problem of antibiotic-resistant microorganisms results in an urgent need for substitutes to conventional antibiotics with novel modes of action and effective activities. Antimicrobial peptides (AMPs), produced by a wide variety of living organisms acting as a defense mechanism against invading pathogenic microbes, are considered to be such promising alternatives. AMPs display a broad spectrum of antimicrobial activity and a low propensity for developing resistance. Therefore, a thorough understanding of AMPs is essential to exploit them as antimicrobial drugs. Considering this, we developed a comprehensive user-friendly data repository of antimicrobial peptides (DRAMP), which holds 17349 antimicrobial sequences, including 4571 general AMPs, 12704 patented sequences and 74 peptides in drug development. Entries in the database have detailed annotations, especially detailed antimicrobial activity data (shown as target organism with MIC value) and structure information. Annotations also include accession numbers crosslinking to Pubmed, Swiss-prot and Protein Data Bank (PDB). The website of the database comes with easy-to-operate browsing as well as searching with sorting and filtering functionalities. Several useful sequence analysis tools are provided, including similarity search, sequence alignment and conserved domain search (CD-Search). DRAMP should be a useful resource for the development of novel antimicrobial peptide drugs.

Inappropriate and irrational use of antibiotics has resulted in the emergence of multi-drug resistant microorganisms, spurring an urgent need to develop new generations of antibiotics with novel modes of action and effective activities. Antimicrobial peptides (AMPs), both natural and synthetic which possess mechanisms of antimicrobial activity that are different from those of conventional antibiotics can provide a therapeutic alternative to fight antibiotic-resistant microorganisms^1^. The natural AMPs are isolated and characterized from practically all-living organisms, ranging from prokaryotes to humans^2^. In general, AMPs are small (<10 kDa), cationic and amphipathic molecules with a substantial proportion (≥30%) of hydrophobic residues^3^, thus capable of interacting with microbial membranes through non-specific interactions with the membrane lipids. AMPs display a broad spectrum of antimicrobial activity, being effective against not only gram-positive and gram-negative bacteria, fungi, viruses, protozoa but also insects and some kinds of cancer^4^. Much information suggests that AMPs have major effects on infection and inflammation in mammals by influencing diverse cellular processes^5^. AMPs may act via a range of mechanisms which include, but are not limited to, bacterial membrane disruption^6^, the formation of membrane-spanning pores^7^, the inhibition of cell wall biosynthesis^8^, and translocation across the cytoplasmic membrane to act on intracellular targets^9^. Several antimicrobial peptides or analogues in recent years have been in advanced clinical development for localized or systemic infections^10^,^11^. For example, MX-226 (omiganan pentahydrochloride 1% gel; Migenix), a bovine indolicidin-based peptide, was developed for the prevention of contamination of central venous catheters. In a completed Phase III study, MX-226 demonstrated a significant therapeutic effect on local catheter site infections^12^. Despite those achievements, the therapeutic use of antimicrobial peptides is very limited. Deep understanding of mechanisms of action and the structure-activity relationship of AMPs is necessary to develop new approaches to AMP drugs with improved activity and reduced toxicity. DRAMP is an antimicrobial peptide database created with the objective of providing a useful resource for sequence- and structure-activity studies on AMPs. It currently harbors 17349 entries from extensive literature search and integrates a number of analytical tools to assist researches on AMPs.

## Construction and Content

### Data collection

Antimicrobial peptides in DRAMP were collected from Pubmed, Swiss-prot and Lens^13^ by using keywords such as ‘antimicrobial peptide’, ‘antibacterial peptide’, ‘antifungal peptide’, ‘antiviral peptide’, ‘antitumor peptide’, ‘anticancer peptide’, ‘antiparasitic peptides’ or ‘insecticidal peptide’. The hits were registered into the database if: i) their antimicrobial activities have been demonstrated; ii) the amino acid sequences of peptides have been elucidated; iii) precursor and signal regions have been removed to remain mature sequences; iv) they contain less than 100 amino acid residues. The sequences were divided into General dataset and Patent dataset based on their reference literature. Each entry of General dataset contains following major fields: general field including peptide sequence, length, name, Swiss-prot ID, family, gene, source; activity field containing biological activity, target organisms with MIC values, binding target; structure field including structure type, description, PDB ID; physicochemical field such as Boman index, mass and half-life; literature field including Pubmed ID, journal, author, title; comment field describing detailed biological function of peptides. Patent dataset were annotated with sequence, length, name, source, activity, Patent ID, patent type, publication date, also publication as, title, abstract.

**Figure 1:**
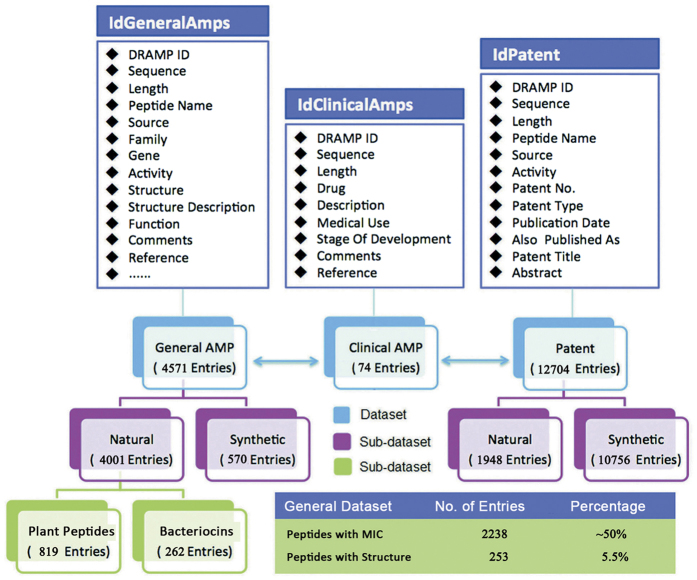
Architecture of the datasets in DRAMP. “IdGeneralAMPs”, “IdClinicalAMPs” and “IdPatentAMPs” are storage structures of the General, Clinical and Patent dataset respectively. The General and Patent datasets fit into two subsets: natural peptides and synthetic peptides. Plant AMPs and bacteriocins belonging to natural AMPs are further stored as separate datasets. About half general peptides in DRAMP are available of MIC values along with target organisms and 253 general entries possess known structures.

Clinical peptides were extracted from literature as separate clinical dataset. The dataset contains 74 antimicrobial peptide entries which have been developed by companies as drug candidates into preclinical or clinical trial stage. Data in this dataset were organized as sequence, name, description, activity, medical use, stage of development, comments, company and reference. It is worthy to note that some clinical peptides whose amino acid sequences are absent were also included in this dataset as we don’t want to lose any clinical information. The architecture of the datasets in DRAMP is shown in Fig. 1.

### Database construction and maintenance

DRAMP was built on Linux platform (32-bits operating system) with Apache web server (version 2.2.22) and MySQL server (v 5.5.29) as the back-end. HTML, PHP and JavaScript was applied to develop the front-end web interfaces. The maintenance of DRAMP contains regular data update, backup, recovery and web optimization.

### Utility

**Figure 2:**
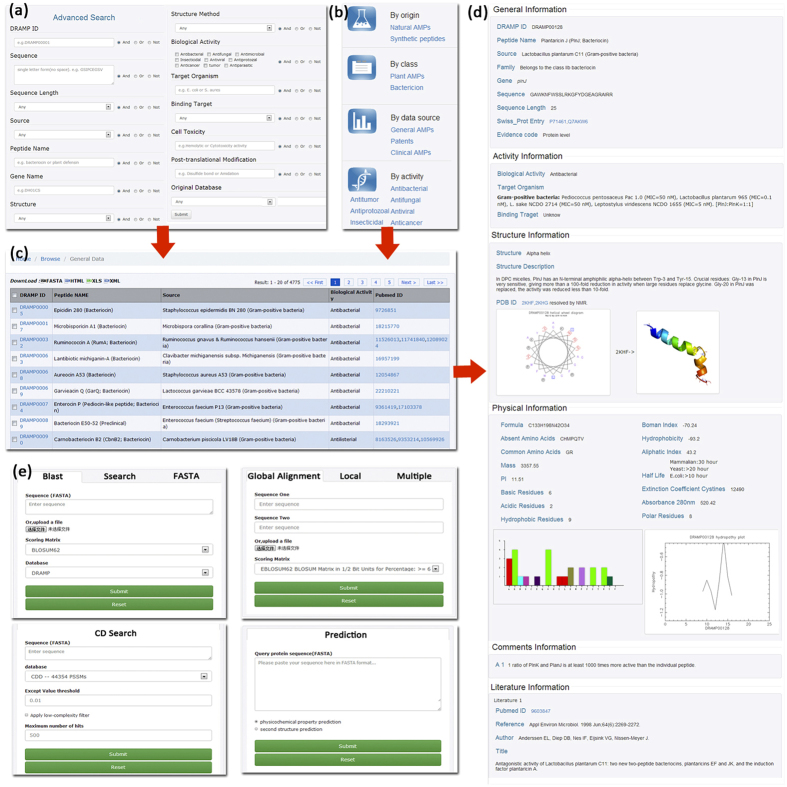
Screenshots of DRAMP web interfaces. (**a**) shows the advanced search page. This interface allows users to query database by a combination of various conditions; (**b**) shows the browse interface of DRAMP; (**c**) shows the result of query or browse; (**d**) shows the detailed information page; (**e**) shows integrated analysis tools in DRAMP.

The main web page of DRAMP contains the following interfaces: Search, Browse, Tools, Statistics, Download and Links. A brief description of the interfaces is given below and the screenshots are given in Fig. 2.

(i)Search page: simple search and advanced search capabilities are constructed in search page though a quick search has been integrated in Home page. Simple search allows users to search the database in a specific field, such as sequence, name or reference, from the drop down menu. Advanced search (Fig. 2 (a)) is a more comprehensive search allowing a combination of keywords like sequence, length, source, peptide name, gene, structure, structure method, biology activity, target organism, binding target, cell toxicity, post-translational modification (PTM) and database ID.

(ii)Browse page: users can browse the database and download sequences that they are interested in. As shown in Fig. 2(b), data can be browsed in General dataset, Patent dataset and Clinical dataset. General data may be further viewed in natural or synthetic sources or in plant AMPs or in bacteriocins. Besides, sequences can be browsed in different activities, viz. antimicrobial, antibacterial, antifungal, antiviral, anticancer, antitumor, antiprotozoal and insecticidal.

(iii)Detailed information page (Fig. 2 (d)): clicking on DRAMP ID of an entry in query or browse results (Fig. 2(c)) can get detailed information page. This page presents all annotations for the entry which are divided into general information, activity information, structure information, physicochemical information, comments information and literature information. In structure part, a helical wheel diagram is shown and if a sequence has the known structure, it can be directly viewed. Besides, amino acid distribution histogram and hydropathy plot are drawn by a sequence’s physicochemical information.

(iv)Tools page: tools integrated in DRAMP include similarity search, alignment search, conserved domain search and physicochemical properties calculator, as Fig. 2(e) shown. Similarity search contains three methods, namely Blast^14^, Fasta^15^ and Ssearch^15^. Blast search can be operated against the local database, or Swiss-prot, or PDB. Alignment search includes global, local and multiple alignment using methods of StretcherP^16^, MatcherP^16^ and Clustal Omega^17^ respectively. Conserved domain search (CD-Search)^18^ allows users to detect structural and functional domains in protein sequences. ProtParam program^19^ and Chou & Fasman algorithm^20^ are integrated for physiochemical properties prediction and protein secondary structure prediction. Users can use the tools by either pasting their peptide sequences in FASTA format or uploading a text file with sequences of their interest.

(v)Statistics page: data statistics of DRAMP are presented in this page, including percentage of amino acids occurrence in AMPs, distribution of peptides length, top 8 AMPs for antimicrobial activity against common microbes in DRAMP.

(vi) Download page: install packages of useful tools such as Blast are collected in this page for download.

(vii) Links page: links of AMP-related databases are collected here for linking to these resources.

### Statistics and findings

**Figure 3:**
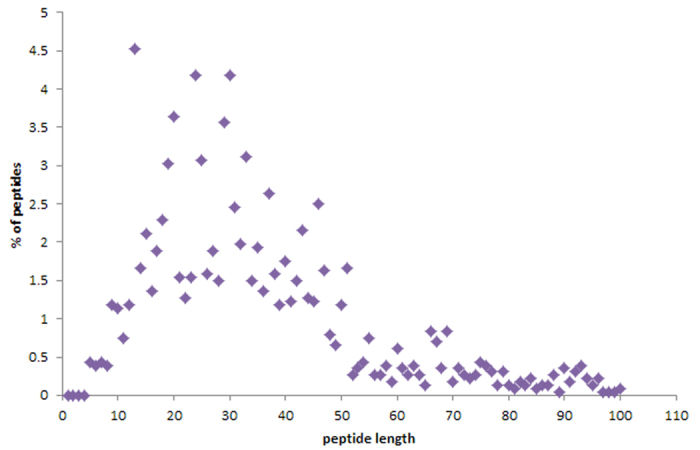
Length distribution of natural antimicrobial peptides. Every scatter indicates the percentage of natural AMPs calculated to have their length ranging from 1 to 100.

**Figure 4:**
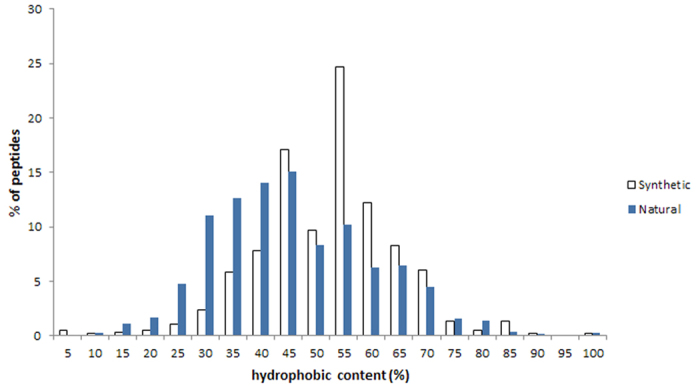
Hydrophobic content distribution in natural and synthetic AMPs. The hydrophobic content is represented as a ratio between the total hydrophobic residues and the total amino acids in a peptide.

**Figure 5:**
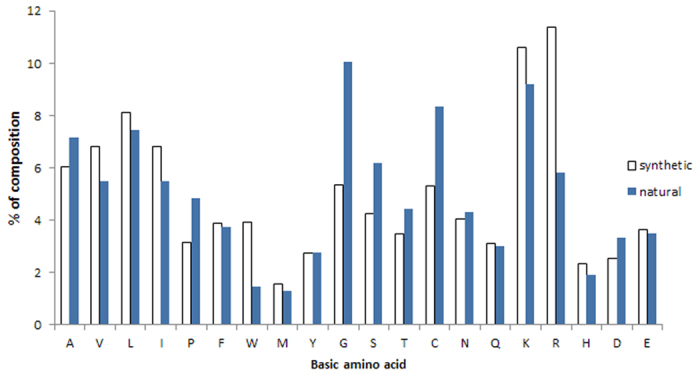
Basic amino acid distribution in natural and synthetic AMPs.

**Figure 6:**
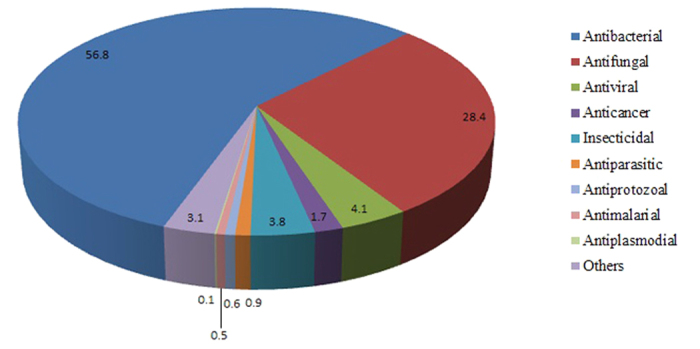
Activity distribution in natural AMPs.

The current version of DRAMP holds 17349 entries in total, which include 4571 general AMPs, 12704 patented sequences and 74 clinical peptides. About half general peptides and 22 clinical peptides are available of MIC values along with target organisms and the top 8 antimicrobial activity are listed in Table 1. 253 general peptides in DRAMP have known structures with PDB ID. To advance our understanding of features of antimicrobial peptides as a basis for peptide design, statistics were carried out in DRAMP. It is found that 90.7% of natural AMPs have a positive net charge with an average value of 3.45 while almost all synthetic AMPs possess positive charges with an average value of 4.78, by analyzing 4001 natural and 570 synthetic sequences in general dataset. 60% natural sequences range from 10 to 50, as can be seen in Fig. 3, while a majority of synthetic peptides have a length of less than 25 amino acids. Figure 4 illustrates the contents of hydrophobic residues in natural and synthetic AMPs. Most of natural peptides possess hydrophobic content in the 30−45% range while most of synthetic peptides range from 45% to 55%. Figure 5 summarizes the basic amino acids distribution. As shown, glycine, cysteine and lysine make up the predominant composition in natural peptides, which is coincident to the statistical result given by APD2^21^ in its website. In contrast, synthetic peptides have a lower ratio of glycine but a higher ratio of arginine. A summary of activities distribution of natural peptides is made in Fig. 6. A specific peptide here may have different activities and thus can be counted twice or more. Most peptides in DRAMP are shown to antibacterial activity (56.8%), followed by antifungal (28.4%). These findings may be useful in developing natural peptide templates or designing novel peptides with improved activities.

## Discussion

### Comparison with other databases

At present, there exist a few databases relating to AMPs, but most of them are specialized to certain categories of AMPs. Some have focused on AMPs produced by plants (PhytAMP)^22^, bacteria (Bactibase)^23^, shrimp (Penbase)^24^ and milk (MilkAMP)^25^, while others have focused on certain properties of AMPs like hemolytic activity (Hemolytik)^26^, activity and structure (DBAASP)^27^, antibacterial activity (YADAMP)^28^, anuran defense peptides (DADP)^29^, antiviral activity (AVPdb)^30^ and anti-HIV activity (HIPdb)^31^. Those databases have played an important role in certain categories of AMPs but have limits on sequences collection. To the best of our knowledge, there exsit four databases dealing with AMPs from diverse origins and they are APD2^21^, DAMPD^32^, CAMP^33^ and LAMP^34^. APD2 is the most popular public collections of AMPs, harboring 2625 mature sequences. DAMPD is a replacement of the ANTIMIC^35^ database and has extended its entries to 1232, including both precursor and mature sequences. CAMP holds 8164 entries (experimentally validated (2774) and predicted (5390)) and has integrated an antimicrobial activity prediction function based on machine learning algorithms. LAMP is a cross-linking database providing hyperlinks to other databases. Compared to these databases, DRAMP holds diverse annotations of AMPs including sequence information, structure information, physicochemical information, patent information, clinical information, reference information and especially antimicrobial activity information (shown as target organisms with MIC values). Moreover, multiple tools are available on the database for sequence analysis. DRAMP is expected to be a useful resource for rational design of novel antimicrobial peptides.

### Future directions

The physicochemical properties and activity relationship extracted from the collection of AMPs has proved to be a powerful resource for the design of novel antimicrobial peptides. The Nebraska Medical Center has carried out the design of anti-MRSA peptides based on peptide information in the APD database^36^. SVM-based activity prediction tools have been implemented in CAMP and C-PAmP^37^. We will conduct more data mining and design work based on our collection of AMPs in the future. For example, novel peptides are being developed based on 121 experimentally validated anti-MRSA peptides^38^.

## Conclusion

DRAMP is an open-access, manually curated database aiming at making a comprehensive repository of AMPs. DRAMP mainly holds three datasets, general, patented and clinical dataset. Activity information (MIC values) and structure information are manually collected from the literature. Clinical entries with pharmaceutical information are registered in clinical dataset. User-friendly interfaces have been established to facilitate peptides searching, browsing and alignment. DRAMP should help promote our understanding of antimicrobial peptides and should provide a valuable resource for the development of novel drugs.

### Availability and requirements

DRAMP can be accessed freely at http://dramp.cpu-bioinfor.org.

## Additional Information

**How to cite this article**: Fan, L. *et al.* DRAMP: a comprehensive data repository of antimicrobial peptides. *Sci. Rep.* **6**, 24482; doi: 10.1038/srep24482 (2016).

## Figures and Tables

**Table 1:** Top 8 AMPs for antimicrobial activity in DRAMP.

Class	Microbes	MIC (μg/ml)	DRAMP ID
Gram-positive bacteria	*S. aureus*	0.0009	DRAMP02470
	*S. aureus* (MRSA)	0.047	DRAMP00204
	*M. lysodeikticus*	0.02	DRAMP03471
Gram-negative bacteria	*P. multocida*	0.0024	DRAMP02470
	*P. aeruginosa*	0.01	DRAMP04069
	*E. coli*	0.02	DRAMP00191
Fungi	*C. jejuni*	0.025	DRAMP00089
	*C. albicans*	0.16	DRAMP03580
